# Advancements, challenges, and future prospects in clinical hyperpolarized magnetic resonance imaging: A comprehensive review

**DOI:** 10.1016/j.bj.2024.100802

**Published:** 2024-10-21

**Authors:** Ching-Yi Hsieh, Ying-Chieh Lai, Kuan-Ying Lu, Gigin Lin

**Affiliations:** a Research Center for Radiation Medicine, Chang Gung University, Taoyuan, Taiwan; b Department of Medical Imaging and Radiological Sciences, Chang Gung University, Taoyuan, Taiwan; c Department of Medical Imaging and Intervention, Chang Gung Memorial Hospital at Linkou, Taoyuan, Taiwan; d Clinical Metabolomics Core Laboratory, Chang Gung Memorial Hospital at Linkou, Taoyuan, Taiwan

**Keywords:** Carbon-13, Dynamic nuclear polarization, Hyperpolarization, Magnetic resonance imaging, Magnetic resonance spectroscopy

## Abstract

Hyperpolarized (HP) magnetic resonance imaging (MRI) is a groundbreaking imaging platform advancing from research to clinical practice, offering new possibilities for real-time, non-invasive metabolic imaging. This review explores the latest advancements, challenges, and future directions of HP MRI, emphasizing its transformative impact on both translational research and clinical applications. By employing techniques such as dissolution Dynamic Nuclear Polarization (dDNP), Parahydrogen-Induced Polarization (PHIP), Signal Amplification by Reversible Exchange (SABRE), and Spin-Exchange Optical Pumping (SEOP), HP MRI achieves enhanced nuclear spin polarization, enabling in vivo visualization of metabolic pathways with exceptional sensitivity. Current challenges, such as limited imaging windows, complex pre-scan protocols, and data processing difficulties, are addressed through innovative solutions like advanced pulse sequences, bolus tracking, and kinetic modeling. We highlight the evolution of HP MRI technology, focusing on its potential to revolutionize disease diagnosis and monitoring by revealing metabolic processes beyond the reach of conventional MRI and positron emission tomography (PET). Key advancements include the development of novel tracers like [2–^13^C]pyruvate and [1–^13^C]-alpha-ketoglutarate and improved data analysis techniques, broadening the scope of clinical metabolic imaging. Future prospects emphasize integrating artificial intelligence, standardizing imaging protocols, and developing new hyperpolarized agents to enhance reproducibility and expand clinical capabilities particularly in oncology, cardiology, and neurology. Ultimately, we envisioned HP MRI as a standardized modality for dynamic metabolic imaging in clinical practice.

## Graphical abstract

The flow of 13C substrate preparation, HP 13C polarization, MRI acquisition, and reconstruction. This diagram delineates the comprehensive process of employing HP 13C substrates, like pyruvate, to visualize downstream metabolites—lactate, alanine, and bicarbonate—in the glycolysis pathway through MRI.

**Figure:**
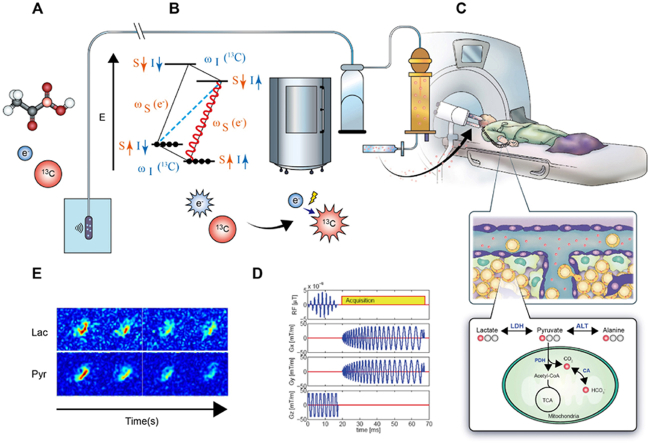

## 1 Introduction

Hyperpolarized (HP) magnetic resonance imaging (MRI) represents a groundbreaking advancement in medical imaging, uniquely positioned between the conventional MRI and positron emission tomography (PET). Unlike conventional MRI, which visualizes anatomical structures using 1H nuclei, HP MRI uses labeled compounds with significantly enhanced nuclear spin polarization [1]. This results in a dramatic increase in signal intensity, allowing for real-time observation of metabolic processes in the body. In contrast to PET-FDG (18F-fluorodeoxyglucose), which tracks glucose metabolism using radioactive tracers, HP 13C MRI is non-radioactive and provides a different metabolic perspective. While PET-FDG is excellent for identifying areas of high glucose uptake, often correlating with tumor activity, it cannot distinguish between different metabolic pathways. HP 13C MRI, on the other hand, can provide detailed information on specific metabolic pathways, such as Krebs cycle activity in tumors, offering deeper insights into cellular metabolism and function [2,3] and the potential for early detection of treatment effects.

Even though this technique has the potential to reveal unprecedented metabolic insights, it also presents challenges, particularly in the realms of data acquisition, post-processing, data analysis, and the use of tracer [1–^13^C]pyruvate. These challenges stem from the short-lived nature of the hyperpolarized signal and the complexity of interpreting metabolic data. This review primarily describes the techniques and methods developed based on dissolution dynamic nuclear polarization (dDNP) and aims to address these challenges which involve developing advanced techniques for rapid and efficient data acquisition, sophisticated algorithms for post-processing to maximize signal extraction, and robust methods for data analysis to accurately interpret metabolic pathways. The use of [1–^13^C]pyruvate as a tracer plays a crucial role in this context, as it is pivotal in studying key metabolic processes, especially in cancer cells.

Building on this foundation, it is essential to explore the unique roles of HP 13C pyruvate in both translational research and clinical practice. In translational research, HP 13C pyruvate MRI has proven invaluable for understanding disease mechanisms, evaluating new therapies, and identifying biomarkers. For example, this technique is employed to study fundamental metabolic changes associated with diseases such as cancer [4,5], cardiovascular conditions [6], and neurological disorders such as mild Traumatic Brain Injury (TBI) [7,8], and hypoxic-ischemic encephalopathy (HIE) [9]. Researchers utilize HP 13C pyruvate MRI to visualize how diseases alter metabolic pathways, such as the conversion of pyruvate to lactate in cancer cells (Warburg effect), leading to the discovery of novel therapeutic targets and the development of new treatment strategies [10]. Additionally, in preclinical studies, HP 13C pyruvate MRI is used to assess the metabolic effects of experimental drugs or interventions, such as monitoring how a new cancer drug affects lactate production in tumor models, thus identifying potential therapeutic benefits or toxicities early in the drug development process [11,12]. The technique also facilitates the discovery of new metabolic biomarkers that indicate disease presence, progression, or response to treatment, such as changes in lactate production that correlate with tumor aggressiveness [13].

In clinical practice, HP 13C pyruvate MRI is applied directly to patients to diagnose disease, guide treatment decisions, monitor therapeutic response, and evaluate disease progression. For example, it allows for the detection of metabolic alterations in prostate cancer patients before structural changes are visible on conventional imaging, enabling earlier diagnosis and intervention [[14], [15], [16]]. Clinicians can tailor treatment plans based on individual metabolic profiles, assessing tumor activity to determine a patient's likelihood of responding to chemotherapy or radiation [17]. Moreover, HP 13C pyruvate MRI offers the advantage of detecting metabolic changes that occur much earlier than changes in tumor size, helping to quickly identify whether a treatment is effective or if a change in strategy is needed. It also enables the non-invasive tracking of disease progression over time, which is particularly valuable in conditions like cancer diseases [14,16,17], and neurological disorders [18], where early detection of progression can significantly impact clinical management.

Building on these roles, the manuscript will also present a comparative analysis of three alternative HP techniques: parahydrogen induced polarization (PHIP), spin exchange optical pumping (SEOP), and signal amplification by reversible exchange (SABRE). Each of these methods offers distinct approaches to enhancing nuclear spin polarization, thus broadening the scope of HP 13C applications.

## 2 HP 13C MRI: Technical advancements and unmet need

Dissolution dynamic nuclear polarization (dDNP) significantly enhances the capabilities of HP 13C imaging, a technique at the forefront of metabolic imaging. At its core, dDNP involves a process where 13C-labeled compounds are mixed with an electron-rich free radical [1]. This mixture is then cooled to extremely low temperatures, close to 1 Kelvin, within a high magnetic field. The purpose of this cooling step is to align the spins of the electrons in the free radicals to a high degree. Once this high level of electron spin polarization is achieved, microwave irradiation is applied. The microwave with specific frequencies target the interaction between the electron spins of the free radicals and the nuclear spins of the 13C nuclei. Through this interaction, polarization is transferred from the electrons to the 13C nuclei. This transfer dramatically increases the nuclear spin polarization of the 13C atoms, by a factor of 10,000 to 100,000 times greater than what is normally achieved in conventional MRI. This boosted polarization results in a significantly enhanced signal when these atoms are observed in an MRI scanner.

**Fig. 1:**
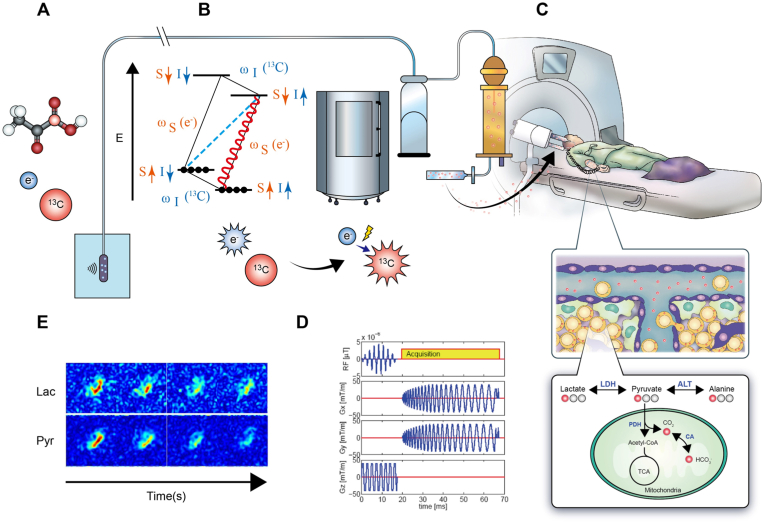
The flow of 13C substrate preparation, HP 13C polarization, MRI acquisition, and images. This diagram delineates the comprehensive process of employing HP 13C substrates, like pyruvate, to visualize downstream metabolites—lactate, alanine, and bicarbonate—in the glycolysis pathway through MRI. Initially, the 13C substrate undergoes preparation in a sterile environment, setting the stage for its transformation into a highly informative metabolic tracer (A). Subsequently, the substrate is subject to polarization via the DNP technique such as GE SPINlab (B).

After the DNP process, the hyperpolarized substrate undergoes rapid dissolution in a biocompatible solvent. The liquid form of the substrate is delivered by the sterile fluid path [19]. This step is critical and time-sensitive, as the enhanced polarization of the 13C nuclei begins to decay quickly, typically within a few minutes. The dissolved, hyperpolarized agent is then ready for injection and subsequent imaging. This unique capability of dDNP to create a highly polarized state in 13C-labeled compounds is what allows for the real-time observation of metabolic processes in biological tissues, providing unprecedented insights into cellular metabolism and function. The schematic process is illustrated in [Fig. 1]. Utilizing 13C fast data acquisition and dDNP technique, these images have revealed the metabolic alterations in the human prostate [14], brain [20], breast [21], kidney [22], heart [23], as well as the immune system, both in both preclinical [24] clinical studies. Although the real-time metabolic imaging technique is promising, there are still challenges in imaging acquisition, data analysis, and the substrate of [1–^13^C]pyruvate. We will discuss these in the following sessions.

## 3 Comparison of dDNP with other hyperpolarization techniques

Parahydrogen-induced polarization (PHIP): PHIP utilizes hydrogen gas enriched in its para-spin state to hyperpolarize substrates before their incorporation into molecules of interest [25,26]. This method is cost-effective and allows for significant signal enhancement but is constrained by its requirement for a hydrogenation reaction, limiting its applicability to molecules that can undergo such a process. PHIP's application has predominantly been within preclinical studies due to its specific requirements for substrate modification and the need for further investigation into its clinical viability [27].

Signal amplification by reversible exchange (SABRE): SABRE represents a versatile hyperpolarization technique employing catalysts to transfer polarization from para-hydrogen to target nuclei, including protons [[28], [29], [30]]. This method has enabled the hyperpolarization of various compounds, such as [1–^13^C]- and [2–^13^C]pyruvate [31,32], and even [1–^13^C]-alpha-ketoglutarate, showcasing its capability to produce long-lived hyperpolarized states [33]. SABRE's adaptability and the ability to hyperpolarize a wide array of nuclei suggest its potential for both preclinical research and clinical applications, pending further development and validation [34].

Spin-exchange optical pumping (SEOP): HP 129Xenon (Xe) MRI, enabled by the SEOP technique, represents a significant advancement in non-invasive lung imaging. This method involves polarizing 129Xe gas in a heated cell containing rubidium vapor under a low magnetic field, where circularly polarized laser light induces spin polarization transfer from rubidium electrons to 129Xe nuclei [35]. Achieving polarization levels between 20% and 50%, SEOP greatly enhances MRI signal sensitivity compared to conventional techniques. HP 129Xe MRI has been safely applied in clinical studies [[36], [37], [38], [39]], improving the visualization of lung ventilation and gas exchange without the use of ionizing radiation. Exploiting the unique chemical shifts of 129Xe in different lung compartments allows for a detailed assessment of lung function, including ventilation defects and gas exchange efficiency [38,40,41]. This technique has proven particularly valuable in identifying and quantifying ventilation impairments, using both reader-based trials and semi-automated binning approaches for accurate ventilation defect percentage (VDP) calculation [[42], [43], [44], [45]]. Furthermore, HP 129Xe MRI facilitates the study of lung microstructures through diffusion-weighted imaging, enhancing our understanding of diseases like chronic obstructive pulmonary disease (COPD) [46]. The safe and effective use of HP 129Xe MRI underscores its potential for advancing respiratory diagnostics and research, offering a powerful tool for exploring lung physiology and pathology with unprecedented clarity and precision. [Table 1] summarizes these techniques and lists the pros and cons of each technique.

**Table 1:** Comparison of hyperpolarization techniques.

HP Technique	Principle	Primary substrate	HP Duration	Advantages	Challenges	References
dDNP	Polarization transfer from electron spins to 13C nuclei at low temperatures and high magnetic fields	[1–13C]pyruvate	∼120 min	High polarization levels, real-time metabolic imaging	Requires cryogenic temperatures, and long polarization time	[1,14,[19], [20], [21], [22], [23]]
PHIP	Hydrogen gas enriched in para-spin state used to hyperpolarize substrates	Substrates suitable for hydrogenation	Minutes	Cost-effective, significant signal enhancement	Limited applicability due to hydrogenation requirement	[[25], [26], [27]]
SABRE	Use of catalysts to transfer polarization from para-hydrogen to target nuclei	Various compounds, including [1–13C]- and [2–13C]pyruvate	Minutes	Versatile, capable of hyperpolarizing a wide array of nuclei	Need for further development and validation	[[28], [29], [30], [31], [32], [33], [34]]
SEOP	Polarization transfer from rubidium electrons to 129Xe nuclei using circularly polarized laser light	129Xenon	Minutes	Non-invasive lung imaging, chemical shift between blood and tissue, safe for clinical studies	Lower polarization efficiency, and limited clinical applications	[[35], [36], [37], [38], [39], [40], [41], [42], [43], [44], [45], [46]]

Abbreviations: dNDP: dissolution dynamic nuclear polarization; PHIP: parahydrogen induced polarization; SEOP: spin exchange optical pumping; and SABRE: signal amplification by reversible exchange.

In comparison, while PHIP offers a cost-effective approach, its utility is somewhat limited by the specificity of its substrate requirements. SABRE, on the other hand, showcases broader applicability with the ability to hyperpolarize a wide range of nuclei, positioning it as a promising technique for both preclinical and potentially clinical applications. SEOP distinguishes itself by its clinical adoption in lung imaging, facilitated by the direct use of hyperpolarized noble gases. Collectively, these techniques exemplify the ongoing evolution and diversification of hyperpolarized MRI methods, each contributing uniquely to the expansion of imaging capabilities in both research and clinical domains.

## 4 Challenges and remedies in imaging techniques

HP 13C MRI, a transformative imaging technique, enables the non-invasive observation of metabolic pathways by enhancing the signal of 13C-labeled substrates. This advancement illuminates the complex biochemical processes within living tissues, offering significant potential in patient diagnosis and treatment monitoring. However, its broader application is hindered by various technical and practical challenges, necessitating innovative solutions to maximize its clinical utility and unlock its full potential.

One primary challenge is the limited 13C imaging window due to the short-lived nature of the hyperpolarized signal. To address this, researchers have developed advanced pulse sequences and imaging techniques that optimize signal utilization within this constrained timeframe. For example, flyback echo-planar spectroscopic imaging (EPSI) and symmetric EPSI have improved data acquisition efficiency [47,48], while rapid 3D volume imaging techniques using spectral-spatial excitation pulse (SPSP) with echo-planar imaging (EPI) or spiral readouts have enhanced the signal-to-noise ratio (SNR) of metabolites [49,50]. Additionally, the iterative decomposition with echo asymmetry and least squares estimation (IDEAL) chemical shift imaging (CSI) offers an efficient method for decomposing multiple chemical species [51,52].

**Fig. 2:**
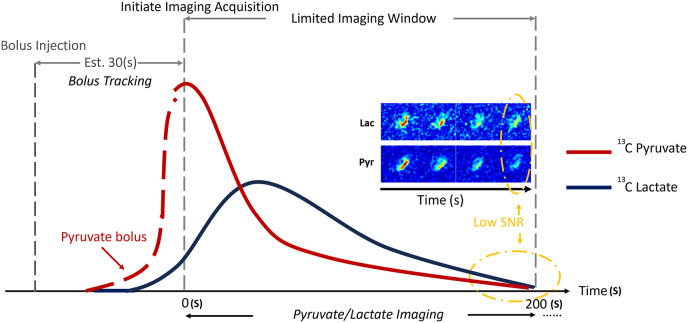
Challenges in HP 13C imaging acquisition and data analysis. This diagram vividly depicts the dynamic nature and inherent challenges of HP 13C imaging, focusing on the temporal progression of HP 13C pyruvate (red) and its metabolite lactate (blue) signals from injection up to 200 s, when signals diminish into the noise threshold. The depiction underscores the unique attribute of flux in HP 13C imaging, where the degrading metabolic signals over time highlight critical obstacles. The primary challenge illustrated is the limited imaging window, constrained to less than 200 s, demanding precise timing for optimal signal capture. The second challenge involves determining the optimal acquisition time within this narrow window to maximize data quality and relevance. Thirdly, the diagram points to the complexity of defining the pyruvate flow function, a pivotal component in the kinetic model necessary for estimating apparent exchange rate constants, crucial for accurate metabolic analysis. Lastly, the rapid degradation of the hyperpolarized state presents a significant hurdle, leading to a decreased signal-to-noise ratio (SNR) towards the end of the acquisition period. These challenges highlight the technical and temporal intricacies of HP 13C imaging, underscoring the need for precise timing and sophisticated modeling to harness its full diagnostic potential.

Prescan procedure complexities, arising from the small amount of natural 13C abundance, complicate automated settings like shim parameters and RF transmit gain. Remedies include utilizing Sodium 23 or Hydrogen 1 frequencies for 13C pyruvate frequency estimation and calibrating transmit gain with a 13C Urea phantom using Bloch-Siegert pulses [53,54]. Variability in HP 13C pyruvate bolus due to physiological differences is mitigated by innovative bolus tracking methods and frameworks for simultaneous automatic acquisition timing and B1 calibration to adjust for variability [55,56]. These challenges are illustrated in [Fig. 2].

Challenges in achieving high spatial and temporal resolution in dynamic environments overlap with the limited imaging window issue. Using multiband excitation pulses in parallel imaging approaches is one way to address this issue. The other method involves using compartmental information from the matching high-resolution 1H images to improve the spatial resolution of HP 13C human organ images using a patch-based algorithm [13,[57], [58], [59]]. Furthermore, the application of balanced steady-state free-precession (bSSFP) sequences for the recurrent refocusing of transverse spins enhances the spatiotemporal resolution of metabolites and can also be used to reduce respiratory motion aberrations in abdominal imaging [60,61]. Lastly, addressing the production and delivery challenges of hyperpolarized agents involves investigations into cost-effective production methods and the development of preservation techniques to extend the duration of the enhanced signal, tackling issues related to cost, availability, efficient delivery, and maintaining hyperpolarization stability [62,63]. These comprehensive solutions not only enhance image quality and reliability but also pave the way for wider clinical adoption, contributing to improved patient outcomes and deeper understanding of disease mechanisms. [Table 2] summarizes the challenges and corresponding remedies.

**Table 2:** Imaging Techniques of hyperpolarized 13C MRI.

Challenges	Remedies	References
Limited hyperpolarization window	Advanced pulse sequences and imaging techniques: Efficiency in data acquisition, rapid 3D volume imaging, IDEAL chemical shift imaging, high-resolution imaging	EPSI [47,48]:
		SPSP [49,50]:
		IDEAL CSI [21,51,52,64,65]:
Prescan procedure complexities	Prescan procedure adaptations: Frequency estimation, transmit gain calibration	Frequency estimation [53]:
		Transmit gain calibration [54]:
Physiological variability in HP 13C pyruvate bolus	Bolus tracking enhancements: data acquisition triggering, automatic acquisition and calibration	Data acquisition triggering [55]:
		Automatic acquisition and calibration [56]:
Spatial and temporal resolution	Compressed sensing and multiband excitation pulses, utilization of 1H image by using patch-based algorithm	Spatial and temporal resolution [13,[57], [58], [59]]:
Respiratory motion in abdominal imaging	Mitigation of respiratory motion artifacts by fast acquisition	bSSFP [60,61]:
Production and delivery of hyperpolarized agents	Investigations into cost-effective production methods and preservation techniques	Production and preservation techniques [62,63]:

Abbreviations: EPSI: flyback echo-planar spectroscopic imaging; SPSP: spectral-spatial excitation pulse; IDEAL: iterative decomposition with echo asymmetry and least squares estimation; CSI: chemical shift imaging; bSSEP: balanced steady-state free-precession.

## 5 Challenges and remedies in data processing and analysis

HP 13C MRI plays a critical role in unveiling dynamic metabolic processes, providing invaluable insights into alterations in glycolytic flux. This advanced imaging technique, through spectroscopy and imaging, offers a detailed view of metabolic fluxes, which are essential for understanding disease mechanisms and evaluating treatment responses. Accurate data analysis is pivotal in interpreting these fluxes, either through direct measurement using the area under the curve (AUC) method [66] or via kinetic modeling [[67], [68], [69]], presenting the complexities and nuances of metabolic activities within the body.

The kinetic model for analyzing HP 13C MRI data accounts for the flow of HP 13C pyruvate into an organ and its chemical exchange with lactate. This model is described by differential equations that consider the relative signal intensities of pyruvate, P(t) and lactate, L(t), the incoming pyruvate signal intensity, u(t), reaction rate constants, K_P_ and K_L_, and effective relaxivity, ρ, in Equations (1), (2)). Such modeling enables the quantification of metabolic fluxes, including the conversion rate of pyruvate to lactate, which is indicative of metabolic activity and alterations in diseases.

(1) $$\;\;\;\;\;\;\;\;\;\;\;\;\;\;\;\;\;\;\;\;\frac{\text{dP}\left(t\right)}{\text{dt}}=k_{L}L\left(t\right)‐\left(k_{P}+\rho \right)P\left(t\right)+u\left(t\right)$$

(2) $$\frac{\text{dL}\left(t\right)}{\text{dt}}=k_{p}P\left(t\right)‐\left({\;k}_{L}+\rho \right)L\left(t\right)$$

where P and L are the relative pyruvate and lactate signal intensities, and u is the pyruvate incoming signal intensity, K_p_ and K_l_ are the forward and backward reaction rate constants, respectively, and ρ is the effective relativity given by

(3) $$\rho =\frac{1}{T_{1\;\text{eff}}}=\frac{1}{T_{1}}‐\frac{1}{t_{R}}\ln\left(\cos\;\theta \right)$$

where T_1_ is the relaxation time of pyruvate in the medium or tissue, t_R_ is the repetition time, and θ is the flip angle.

Addressing the challenges in this analysis requires innovative approaches. For instance, the estimation of Pyruvate Flow (u(t)) in the kinetic model is challenging due to short imaging windows and the limited signal of HP 13C pyruvate. Solutions include the use of Gd-DTPA in dynamic contrast-enhancement (DCE) MRI to estimate u(t), and co-polarization techniques of 13C urea and pyruvate for simultaneous perfusion and metabolic information, respectively [13,[70], [71], [72]]. The alternative approach is to focus solely on fitting the lactate signal, using the measured pyruvate signal as the input, u(t), for the kinetic model at every time point. This method involves fitting the estimated lactate signal at each time point based on the pyruvate signal measured at adjacent time points and the previously estimated lactate signal. This is achieved by applying the two-site model described in differential Equations (1), (2)), thereby refining the model's accuracy in capturing the dynamics between lactate and pyruvate signals [69].

The other challenge is the low signal-to-noise ratio (SNR) of 13C downstream metabolites, which can cause discrepancies in AUC results or Kp values. Remedies include denoising of HP 13C spectroscopy and images using singular value decomposition (SVD)-based low-rank methods, applying high-order SVD with low rank or wavelet methods for image denoising, and utilizing kinetic models with background noise features for accurate signal estimation of downstream metabolites [[73], [74], [75]]. The challenges of data acquisition and analysis are illustrated in [Fig. 2].

Bridging the gap between the aforementioned challenges is the overarching issue of reproducibility in 13C data analysis. Ensuring consistent outcomes across different hyperpolarized 13C MRI studies is paramount, as variability in data analysis can undermine the reliability of research findings. The introduction of the Spectroscopic Imaging, Visualization, and Computing (SIVIC) open-source software package marks a significant step towards enhancing reproducibility [76]. Furthermore, the availability of analytical tools for the kinetic model through the “Hyperpolarized MRI Toolbox” [77] via the Hyperpolarized Technology Resource Center represents an invaluable resource for researchers. By addressing the challenges of reproducibility, the scientific community can advance towards more consistent and reliable analysis in hyperpolarized 13C studies, thereby facilitating the translation of research findings into clinical applications.

These strategies highlight the concerted efforts to overcome data processing and analysis challenges in HP 13C MRI, aiming to refine techniques for a better understanding of metabolic alterations. Through such advancements, researchers and clinicians can gain deeper insights into the metabolic pathways, facilitating improved diagnosis, monitoring, and treatment of diseases. [Table 3] summarizes the challenges and corresponding remedies.

**Table 3:** Data analysis of HP 13C MRI.

Challenges	Remedies	References
Estimating pyruvate flow (u(t))	1 DCE MRI integration with kinetic and perfusion models 2 Fitting the apparent exchange rate using the measured pyruvate signal 3 Co-polarization of 13C urea and pyruvate for simultaneous perfusion and metabolic information	DCE MRI [70]: Kinetic model equations [69]: 13C urea and pyruvate co-polarization [13,71,72]:
Signal-to-noise ratio (SNR) discrepancy in sequential acquisition	1 Denoising using SVD-based low-rank methods 2 Application of high-order SVD with low rank or wavelet methods 3 Utilization of kinetic models with background noise features	SVD-based methods [73]: High-order SVD and wavelet methods [75]: Kinetic models with noise features [74]:
Reproducibility	1 Open-source SIVIC software package 2 Open-source analytical tools for the HP 13C kinetic model	SIVIC software [76]: HP 13C Kinetic model [77]:

Abbreviations: DCE: dynamic contrast-enhancement; SVD: singular value decomposition; SNR: signal-to-noise ratio; SIVIC: Spectroscopic Imaging, Visualization, and Computing.

## 6 Advances in metabolic imaging: Overcoming limitations with novel 13C-labeled tracers and co-polarization techniques

HP [1–^13^C]pyruvate has been a cornerstone in metabolic imaging, offering critical insights into cellular metabolism and disease processes. However, its scope, particularly in probing Krebs cycle metabolites and assessing glutaminolysis—a crucial pathway in cancer metabolism—has been limited. To address these limitations, significant advancements in tracer technology have been made, expanding the utility of metabolic imaging and overcoming the constraints of [1–^13^C]pyruvate. The development of [2–^13^C]pyruvate, synthesized and polarizable via dDNP, marks a significant advancement by enabling the probing of Krebs cycle metabolites. This substrate has broadened the scope of metabolic imaging, facilitating real-time assessments of metabolism in the heart, brain, and liver across various animal models. Clinical and pre-clinical studies with [2–^13^C]pyruvate have showcased its efficacy in metabolic assessments, revealing its potential in diagnosing and monitoring diseases affecting these organs [[78], [79], [80], [81], [82], [83]]. Similarly, [1–^13^C]-Alpha-Ketoglutarate, another substrate synthesized and polarizable through dDNP, has been instrumental in advancing our understanding of glutaminolysis in cancer cell metabolism. This tracer has facilitated studies on isocitrate dehydrogenase (IDH) activity in various animal models, offering new insights into cancer metabolism and potential therapeutic targets [[84], [85], [86], [87]]. Moreover, innovations in co-polarization techniques, particularly the co-polarization of [13C,15N2]urea and [1–^13^C]pyruvate, have enabled simultaneous metabolic and perfusion imaging. This approach provides comprehensive insights into the interplay between metabolic processes and blood flow, further enhancing the capabilities of metabolic imaging in medical diagnostics [13,71,72]. These advancements in substrate or tracer developments not only surmount the initial limitations associated with HP [1–^13^C]pyruvate but also open new avenues for comprehensive metabolic imaging. The development of [2–^13^C]pyruvate and [1–^13^C]-alpha-ketoglutarate, along with the co-polarization of tracers, represents significant strides in enhancing the scope of metabolic assessments in medical imaging. This progress contributes significantly to the field of medical diagnostics and research, offering new perspectives on disease mechanisms and potential therapeutic interventions. [Table 4] summarizes the characteristics of substrates.

**Table 4:** Tracers beyond HP [1–13C] pyruvate for clinical metabolic MRI.

Tracers	Metabolic Pathway/Function and Applications	References
[2–13C]Pyruvate	Probing Krebs cycle metabolites - applications in heart, brain, and liver metabolism	Krebs cycle analysis [78,83]:
		Brain metabolism [79,80]:
		Diabetic muscles [81]:
		Mouse liver [82]:
[1–13C]-Alpha-Ketoglutarate	Probing metabolic pathways involved in glutaminolysis - instrumental in advancing understanding of cancer cell metabolism	IDH assessment [[84], [85], [86], [87]]:
Co-polarization of [13C,15N2]urea and [1–13C]pyruvate	Allows simultaneous metabolic and perfusion imaging - broadening the scope of metabolic assessments	Simultaneous imaging [13,71,72]:

## 7 Future perspectives

The future perspectives of technical breakthroughs and standardization in HP 13C MRI hold immense promise for advancing the field and facilitating its widespread adoption. One pivotal area of development lies in overcoming existing technical challenges, such as enhancing the duration of hyperpolarization. Technical breakthroughs in hyperpolarization methods, including innovative pulse sequences and hardware improvements, are anticipated to extend the imaging window, allowing for more comprehensive and dynamic imaging of metabolic processes.

Standardization is critical for ensuring consistency and comparability across different hyperpolarized 13C MRI studies. The establishment of standardized protocols for agent production, imaging acquisition, and data analysis is crucial. This standardization would facilitate multi-center collaborations and comparisons, fostering a more robust and reproducible research environment. Currently, several groups have reported the comprehensive survey of HP [1–^13^C]pyruvate in human studies [88]. This is the first step toward the standardization of HP 13C pyruvate. Moreover, future breakthroughs are expected in the development of novel hyperpolarized agents, expanding the range of metabolites that can be studied. This diversification will open new avenues for investigating various metabolic pathways and contribute to a deeper understanding of physiological and pathological processes.

The integration of artificial intelligence and machine learning into hyperpolarized 13C MRI data analysis holds great potential. These technologies can aid in automated image processing, quantitative analysis, and the extraction of meaningful metabolic information, streamlining the workflow and enhancing the clinical applicability of hyperpolarized 13C MRI. One of practical issues is the signal loss or image artifacts caused by respiratory motion. AI models inspired by 1H MRI literature may offer promise in eliminating respiratory motion artifacts [89,90].

In conclusion, the future of hyperpolarized 13C MRI envisions technical breakthroughs that address current limitations, standardization to ensure reproducibility, the development of novel hyperpolarized agents, and the integration of advanced technologies for efficient data analysis. These advancements collectively promise a transformative impact on medical imaging, fostering its evolution into a widely accepted and standardized modality for studying dynamic metabolic processes in vivo.

Upon achieving optimal polarization, the HP 13C substrate is quickly dissolved to maintain its enhanced state and then injected into the patient (B and C). This step is time-sensitive, as the hyperpolarized state degrades rapidly, necessitating swift administration to capture the peak signal. Following injection, MRI acquisition begins, tracking the distribution and metabolic conversion of the substrate into its metabolites within the body's tissues (D).

The culmination of this process is the reconstruction of detailed 13C metabolic images (E). These images provide a unique window into the body's metabolic pathways in real time, offering insights into normal physiology and the alterations that occur in diseases such as cancer. This technique represents a significant advance in medical imaging, enabling the non-invasive study of metabolism and the potential for tailored therapeutic interventions.

## Declaration of competing interest

The authors declare no conflict of interest.

## Data availability statement

The CC BY 4.0 license is a Creative Commons license.

## Author contribution

Conceptualization: Ching-Yi Hsieh, Gigin Lin.

Data curation: Ying-Chieh Lai, Ching-Yi Hsieh.

Formal analysis: Ying-Chieh Lai, Ching-Yi Hsieh.

Funding acquisition: Ying-Chieh Lai, Ching-Yi Hsieh, Gigin Lin.

Investigation: Ching-Yi Hsieh, Gigin Lin.

Methodology: Ching-Yi Hsieh, Gigin Lin.

Project administration: Kuan-Ying Lu.

Resources: Gigin Lin.

Software: Ying-Chieh Lai, Ching-Yi Hsieh.

Supervision: Gigin Lin.

Validation: Ying-Chieh Lai, Ching-Yi Hsieh.

Visualization: Ching-Yi Hsieh.

Writing-original draft: Ching-Yi Hsieh, Gigin Lin.

Writing-review & editing: Ching-Yi Hsieh, Gigin Lin.

## Declaration of generative AI and AI-assisted technologies in the writing process

During the preparation of this work the author(s) used ChatGPT in order to improve the reading and grammar of this manuscript. After using this tool/service, the author(s) reviewed and edited the content as needed and take(s) full responsibility for the content of the publication.

## Funding

This study was funded by 10.13039/100020595National Science and Technology Council, Taiwan (MOST 109- 2628-B-182A-007-, MOST 110-2628-B-182A-018-, MOST 111-2628-B-182A-012-, NSTC 112-2314-B-182A-015-, NSTC 112-2314-B-182A-127-MY3, NSTC 113-2314-B-182A-086) and 10.13039/501100004606Chang Gung Medical Foundation (BMRPF63, SMRPG3K0055, CLRPG3K0024 and CMRPG3M0732).
